# Effects of Zinc Status on Expression of Zinc Transporters, Redox-Related Enzymes and Insulin-like Growth Factor in Asian Sea Bass Cells

**DOI:** 10.3390/biology12030338

**Published:** 2023-02-21

**Authors:** Kanokwan Sansuwan, Orapint Jintasataporn, Lothar Rink, Supawit Triwutanon, Inga Wessels

**Affiliations:** 1 Department of Aquaculture, Faculty of Fisheries, Kasetsart University, Bangkok 10900, Thailand; 2 Institute of Immunology, Faculty of Medicine, University Hospital RWTH Aachen, Pauwelsstr. 30, 52074 Aachen, Germany; 3 Faculty of Veterinary Medicine, Kasetsart University, Kamphaeng Saen, Nakhon Pathom 73140, Thailand

**Keywords:** Asian sea bass, zinc homeostasis, zinc transporters, metallothionein, redox metabolism, insulin-like growth factors

## Abstract

**Simple Summary:**

This article is of special interest for understanding the detriments of zinc deficiency and the benefits of zinc supplementation for aquacultures of Asian sea bass. We found that altering extracellular zinc conditions significantly affected the expression of certain zinc transporters and zinc-binding proteins as well as intracellular free zinc levels in cells from Asian sea bass (*Lates calcarifer*, SF cells). Interestingly, we detected no difference in the effects of organic compared to inorganic zinc supplements, when the same molar concentrations were added to the cultures. Moreover, changes in extracellular zinc conditions impacted on the expression of genes related to the redox balance and to growth hormone metabolism. Our data indicate that zinc deficiency induces a stress response in cells from Asian sea bass, which may be avoided when sufficient amounts of zinc are provided to the cells. We identified SF cells as a suitable model for studies to optimize zinc supplementation in the aquaculture of Asian sea bass.

**Abstract:**

Since Asian sea bass is one of the economically most important fish, aquaculture conditions are constantly optimized. Evidence from feeding studies combined with the current understanding of the importance of zinc for growth and immune defense suggest that zinc supplementation may be a possible approach to optimize aquacultures of Asian sea bass. To investigate the effects of zinc deficiency and zinc supplementation, cells from Asian sea bass were incubated in culture medium with different zinc contents. The expression of genes, important for zinc homeostasis, redox metabolism, and growth hormones was analyzed using RT-PCR. Zinc deficiency induced the expression of certain zinc transporters (ZIP14, ZIP10, ZIP6, ZIP4, ZnT4, ZnT9) as well as of SOD1, IGF I and IGF II, while expression of ZnT1 and metallothionein (MT) was reduced. Zinc supplementation decreased the expression of ZIP10, while expression of ZnT1 and MT were elevated. No differences in the effects of zinc supplementation with zinc sulfate compared to supplementation with zinc amino acid complexes were observed. Thus, extracellular zinc conditions may govern the cellular zinc homeostasis, the redox metabolism and growth hormone expression in cells from Asian sea bass as reported for other fish species. Our data indicate that supplementing aquacultures with zinc may be recommended to avoid detriments of zinc deficiency.

## 1. Introduction

Fish and its products play a major role in human nutrition, providing at least 20% of the protein intake for a third of the world’s population, and the dependence is highest in developing countries [1]. Asian sea bass (*Lates calcarifer*) is one of the economically most important fish in many countries. It is a popular species for aquaculture, because of its fast growth rate, tolerance to a wide spectrum of environmental conditions, and its high demand in domestic and export markets [2,3]. Thus, efficient aquaculture and research on its optimization resulting in a high yield of healthy fish are of major interest [3,4]. In vivo studies on improving culture conditions to maximize output while guaranteeing the health of the fish are ongoing. For example, adjusting the levels of dietary lipids, although fish are only able to utilize these up to a certain level, and at high levels, growth may even be retarded. Alternatively, salinity or water flow rates may be adjusted and optimized [5]. Recently, trace elements were suggested as essential feed additives to optimize the diets for Asian sea bass [3,6]. 

Zinc is an essential trace element required by fish, including Asian sea bass [3]. Zinc promotes growth, amongst others, by altering the expression of growth hormones and factors including insulin-like growth factor (IGF) [7]. Furthermore, zinc plays a vital role in numerous cellular functions including cell proliferation, reproduction, immune function and balancing redox metabolism [8,9]. Changes in zinc homeostasis alter the expression and the activities of redox-related enzymes such as superoxide dismutase (SOD) and catalase (CAT) [7,10]. Moreover, zinc deficiency may result in a reduced growth rate, increased mortality, low body weight, skeletal deformities, cataracts as well as fin and skin erosion in fish. Zinc deficiency was also reported to increase the sensitivity of fish for toxic elements such as arsenic [11]. 

The availability of zinc from the diet depends on the chemical nature of the zinc source and may differ between organic zinc compounds, such as zinc amino acid (ZnAA) complexes, compared to inorganic zinc compounds including zinc sulfate (ZnSO_4_). For example, in abalone, shrimp, channel catfish, rainbow trout and hybrid striped bass organic zinc had a higher bioavailability than inorganic zinc [6,12,13,14,15]. In contrast, studies in tilapia and turbot revealed no significant differences in the availability of zinc from either form [16,17]. Thus, species-dependent differences have been described but the absorption efficiency of zinc from various sources has so far not been characterized for Asian sea bass.

In mammals, zinc homeostasis is maintained by zinc transporting proteins from the solute carrier families Slc39 (Zip) and Slc30 (ZnT) in combination with intracellular and extracellular zinc-binding proteins. Since zinc cannot cross membrane barriers by diffusion, zinc transporters selectively capture and transport zinc ions across biological membranes. At least 14 Zip transporters, which are responsible for transporting zinc into the cytosol, either from extracellular sources or from intracellular organelles, have been identified in mammals. Zinc transporters were found spanning the plasma membrane but also in the endoplasmic reticulum, lysosomes, golgi and other organelles. The 10 identified ZnT transporters act in an opposing manner to the Zip transporters and decrease cytoplasmic zinc levels through transport of zinc from the cytosol to the extracellular space or into organelles [9,18]. In mammals, expression of zinc transporters was found to depend on the cell type, on zinc availability and if the cells are activated. In addition to zinc transporters, zinc-binding proteins, including metallothioneins (MTs), play a central role in maintaining a stable intracellular zinc homeostasis through sequestration or release of zinc [19]. Experiments in zebrafish suggest that, as in mammals, uptake of zinc by fish may be orchestrated by a similar set of zinc transporting and zinc-binding proteins [20,21]. Knowledge on zinc homeostasis in Asian sea bass is still rather rudimentary and is necessary to enable further and deeper research. 

Thus, in this study, the expression and regulation of zinc transporters, depending on the availability of zinc, were tested using a recently established Asian sea bass cell line [22]. In addition, the present study aimed to evaluate the uptake of zinc sulfate (ZnSO_4_) compared to Availa^®^ zinc (ZnAA). Moreover, the role of zinc in redox metabolism and IGF expression in cells from Asian sea bass were investigated. 

## 2. Materials and Methods

### 2.1. Cell Culture

Asian sea bass fry (SF) cells [22] were a kind gift from Prof. Dr. Sek-Man Wong and Prof. Dr. Gen Hua Yue from Temasek Life Sciences Laboratory, National University of Singapore. The culture used consisted predominantly of epithelial-like cells. They were cultured in Leibovitz-15 medium with 20% heat-inactivated fetal calf serum, 2 mM L-glutamine, 100 U/mL potassium penicillin and 100 U/mL streptomycin sulfate (all Sigma-Aldrich, Steinheim, Germany) at 27 °C. For subcultures and before seeding for experiments, cells were washed with phosphate-buffered saline (PBS), trypsinized, rewashed, taken up in fresh medium and diluted to the desired density. For zinc adequate (ZA) conditions, regular medium was used. For zinc supplementation, the medium was adjusted to 50 µM zinc with zinc sulfate (ZnSO_4_, Merck, Darmstadt, Germany) or zinc amino acid (Availa^®^ zinc, ZnAA, Zinpro, Boxmeer, The Netherlands) by adding stock solutions (100 mM) to the medium. To investigate zinc deficiency, cells were cultured in medium, treated with CHELEX resin, where magnesium and calcium (both Merck) were re-supplemented, according to [23] but no zinc was added. Cells were cultured in ZA, ZnSO_4_, ZnAA or CHELEX medium for 3 days, washed with PBS, trypsinized, diluted 1:16 with fresh medium and analyzed 7 days after initial seeding.

### 2.2. Measurement of Free Intracellular Zinc

The uptake of ZnSO_4_ and ZnAA into SF cells was analyzed using the zinc-specific probe FluoZin-3AM (Invitrogen, Karlsruhe, Germany) according to [24]. Cells (ZA) were washed, trypsinized, rewashed, mixed with an equal volume of fresh medium and seeded into 96 well plates (eight wells per treatment) on the day before the experiment. The cells reached 80% confluence at the time of analysis. On the day of the experiment, the adherent cells were washed once with PBS at room temperature. Detachment of the cells was controlled using microscopy. Incubation buffer (100 µL) made 1 µM with FluoZin3-AM was added to each well. Cells were then incubated at 28 °C for 30 min. After incubation, cells were washed once with PBS. Thereafter, 100 µL of measurement buffer were added to the wells. To determine the minimum (F_min_) and maximum fluorescence (F_max_), wells for F_min_ were incubated with 100 µM N, N, N’, N’-tetrakis (-) [2-pyridylmethyl] ethylenediamine (TPEN), while wells for F_max_ were incubated with 100 µM ZnSO_4_ and 50 µM pyrithione. Then, cells were incubated at 28 °C for 15 min and the baseline was recorded. The resulting fluorescence was measured with a Tecan 340 fluorescence multi-well plate reader (Tecan, Crailsheim, Germany) using excitation and emission wavelengths of 485 and 535 nm, respectively. Subsequently, the remaining wells were adjusted to contain 50 µM ZnSO4 or ZnAA by adding stock solutions (100 mM) and a kinetic was recorded. The zinc concentration was then calculated by the formula (KD = 8.9 nM):

(1) $$[\mathrm{Zn}]=\mathrm{KD}\;\times \;\frac{F-F_{min}}{F_{max}-F}$$

### 2.3. Flame Atomic Absorption Spectrophotometry (AAS)

The total zinc content of all media (data not shown) and cells was analyzed by AAS (AAnalyst 800, Perkin Elmer, Baesweiler, Germany). SF cells were washed with PBS, trypsinized, rewashed with wash buffer (0.9% sodium chloride (AppliChem, Darmstadt, Germany), 10 mM ethylenediaminetetraacetic acid (Sigma-Aldrich) and 10 mM N-[2-Hydroxyethyl]piperazine-N’-[2-ethanesulfonic acid] (HEPES, AppliChem)), counted and the pellet of 5 × 10^6^ cells was afterwards lysed in water. Lysate (1 µL) was used to determine the protein content of the samples, using a Bradford assay (Biorad, Germany). The remaining lysate was digested in nitric acid (AppliChem) for 2 h at 80 °C and diluted with water, followed by subsequent zinc measurements with an AAS. Zinc concentrations for cells were normalized to the protein content of the sample. 

### 2.4. Real-Time PCR

Isolation of total RNA and reverse transcription into cDNA were performed, according to the manufacturer’s instructions, using the NucleoSpin RNA II kit (Macherey-Nagel, Dueren, Germany) and the qScript cDNA synthesis kit (QuantaBioscience, Beverly, MA, USA), respectively. Quantitative real-time PCR was performed using SYBR^®^ green reagent and a StepOnePlus Real-Time PCR System (Thermo Fisher Scientific, Darmstadt, Germany).

The relative expression of target genes was determined using a standard curve, established by a mixture of stimulated and unstimulated SF cells. The internal reference control gene was elongation factor-1 (EF-1)A [2] because of its consistent expression level in ZA, ZnSO4, ZnAA or CHELEX cultures. The qPCR primers were as previously published or designed according to the CDS of reference sequences (RefSeq) for the gene of interest including the isoforms or transcript variants found on NCBI [25,26]. Primers were blasted to exclude possible unspecific PCR products. All PCRs were run in duplicate. The qPCR processes were performed as follows: the first step was performed for 15 min at 95 °C, followed by 40 cycles of 15 s at 95 °C for denaturation, and 30–60 s at 50–61 °C for annealing. For Zip10, 30 s at 72 °C for extension were added and water in the PCR mix was replaced by Q Solution (Qiagen, Germany). The third step for the melting curve analysis was performed for 15 s at 95 °C and then 60 s at 60 °C. Product sizes were verified using gel electrophoresis. The primer sequences are summarized in Table 1. For some genes multiple isoforms were available. In that case, all NCBI-RefSeq for isoforms covered by the primers are indicated.

### 2.5. MTT (3-(4,5-Dimethylthiazol-2-yl)-2,5-Diphenyltetrazolium Bromide) Test

SF cells were washed, trypsinized, rewashed with PBS, taken up in ZD, ZA or ZS (50 µM ZnSO_4_) medium, diluted 1:30 in the respective medium and seeded on 96 well plates (8 wells per treatment). After incubating cells for 7 days, MTT-Solution was added until needle-like formazan aggregates were visible. The supernatants were discarded, and dimethyl sulfoxide was added. The plates were shaken for 10 min, and absorbance was measured at 570 nm using 690 nm as reference wavelength. Means of the eight wells were calculated.

### 2.6. Statistics

**Figure 1:**
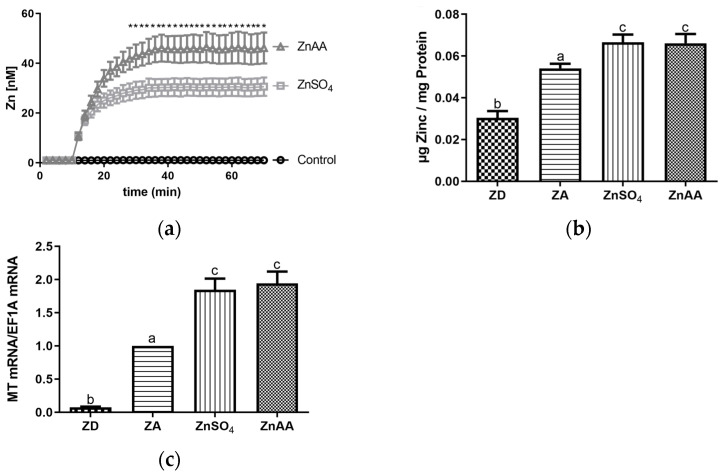
(**a**) A total of 1 × 10^6^ SF cells per ml were loaded with FluoZin3-AM, base line fluorescence was recorded for 10 min and increase in fluorescence after adding ZnSO_4_ (50 µM) or ZnAA (50 µM) was monitored for additional 60 min. (**b**,**c**) SF cells were cultured in CHELEX-treated medium, zinc adequate (ZA) medium or medium supplemented with ZnSO_4_ (50 µM) or ZnAA (50 µM) for 7 days. Afterwards total zinc of the cells was analyzed using AAS or mRNA was isolated and used for measuring MT mRNA expression normalized to EF1A. Mean values + S.E.M. of at least n = 7 independent experiments are shown. Significant differences between (**a**) ZnSO_4_ and ZnAA (multiple *t* tests, * *p* < 0.05) are marked. In (**b**,**c**), significantly different data sets as determined by one-way Anova and Tukey’s post hoc test do not share the same letters (*p* < 0.05).

Outliers were excluded by Grubb’s outlier test and normal distribution of the data was assessed by Shapiro–Wilk normality test. The statistical significance of the results was analyzed by GraphPad Prism software version 9 (GraphPad Software, La Jolla, CA, USA). For comparing two data sets (Figure 1a), multiple paired t-tests were performed, * *p* < 0.05. For comparing more than two data sets, one-way ANOVA with Tukey’s post hoc test was used (*p* < 0.05).

## 3. Results

### 3.1. Zinc Homeostasis in Zinc Deficient and Zinc Supplemented SF Cells

It has been hypothesized that the uptake of zinc into cells strongly depends on the chemical nature of the zinc compound used and that zinc in complex with sulfate, gluconate, aspartate or other amino acids is taken up by cells with differing efficiency. Thus, as a first step, the uptake of zinc sulfate (ZnSO_4_) and a zinc-amino acid complex (ZnAA) into SF cells was analyzed using the zinc-specific probe FluoZin3 AM. As depicted in Figure 1a, ZnSO_4_ and ZnAA were taken up by the cells. Maximum intracellular zinc levels were reached 30 min after adding the supplement. However, the amount of zinc taken up by the cells during this time was significantly higher when ZnAA was added compared to ZnSO_4_ (Figure 1a).

To evaluate if this difference was limited to short incubation times, total cellular zinc was measured 7 days after supplementation was started. As in the short-term incubation, both ZnSO_4_ and ZnAA incubation resulted in a significant increase in total cellular zinc. However, interestingly, no difference was observed at 7 days when total cellular zinc was measured by atomic absorption spectrometry (Figure 1b). In line with this, the expression of metallothionein, the most important intracellular zinc-binding protein, was significantly increased by ZnSO_4_ and ZnAA in the same magnitude (Figure 1c). When cells were cultured in medium where zinc had been depleted using CHELEX resin, total cellular zinc was significantly decreased compared to cells cultured in zinc-adequate conditions (Figure 1b) as was also observed for the expression of metallothionein (Figure 1c).

### 3.2. Zinc Transporter Expression in SF Cells

**Figure 2:**
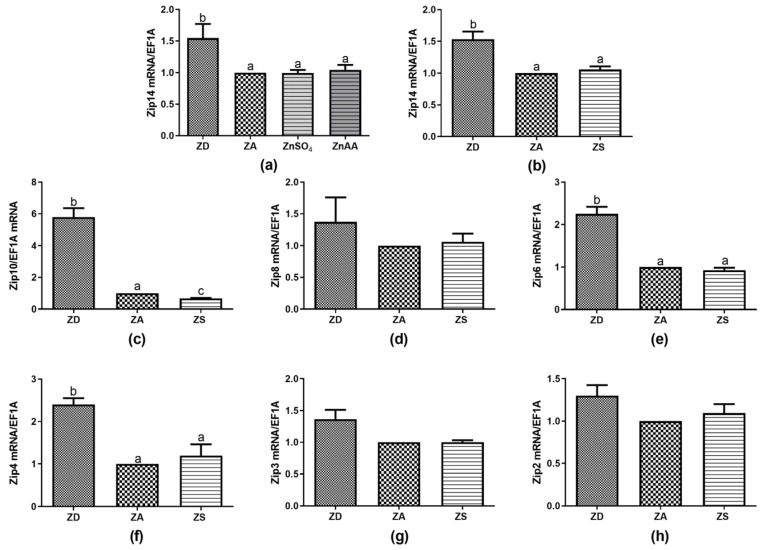
SF cells were cultured in CHELEX-treated medium, zinc adequate (ZA) medium or medium supplemented with ZnSO_4_ (50 µM) or ZnAA (50 µM) for 7 days. Afterwards mRNA was isolated, used for measuring expression of Zip14 (**a**,**b**), Zip10 (**c**), Zip8 (**d**), Zip6 (**e**), Zip4 (**f**), Zip3 (**g**), Zip2 (**h**) mRNA expression and normalized to EF1A mRNA. Mean values + S.E.M. of at least n = 4 independent experiments are shown. Significantly different data sets as determined by one-way Anova and Tukey’s post hoc test do not share the same letters (*p* < 0.05).

Aiming at a more detailed characterization of zinc homeostasis and at finding an explanation for the changes in the zinc status of SF cells, the expression of zinc importing transporters was analyzed. No data on the expression of zinc transporters in cells from the Asian sea bass were available to date. Figure 2 summarizes the results for transcription of those transporters that were found to be expressed by SF cells. However, the expressions of Zip1, Zip7, Zip9, Zip11, Zip12 and Zip13 were below the detection limit and no homologous sequence was found in NCBI for Zip5 (Table 2).

No significant difference was found between the expression induced by ZnSO_4_ compared to ZnAA, as exemplified for Zip14 (Figure 2a). Thus, data for ZnSO_4_ and ZnAA supplementation were averaged and are subsequently summarized as zinc supplementation (ZS). Interestingly, ZD cells showed higher expression levels for all the Zip mRNAs detected than in ZA or ZS cells, with some Zips, e.g., Zip14, Zip10, Zip6, and Zip4 being more strongly affected than others (Figure 2b,c,e,f). Zinc supplementation significantly decreased the expression of Zip10 in comparison to ZA controls. There was no statistically significant difference between the ZA or ZS cells for any of the other detected Zips (Figure 2b–f). However, some of the transporters, including Zip2, Zip4 and Zip14 showed some increase in expression in zinc supplemented compared to zinc adequate cells.

**Figure 3:**
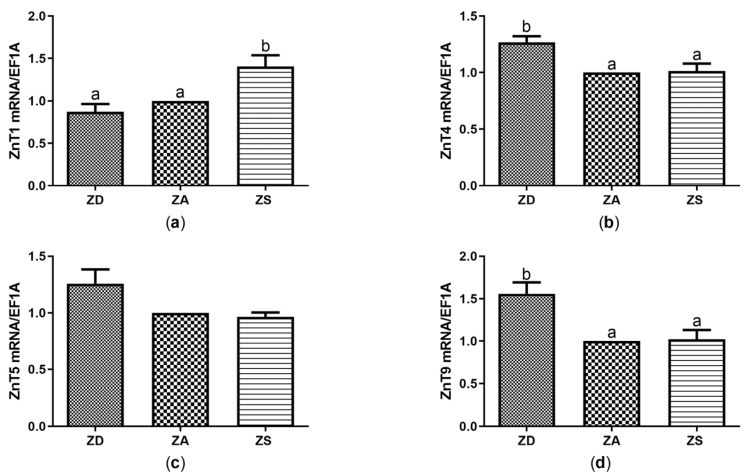
SF cells were cultured in CHELEX-treated medium, zinc adequate (ZA) medium or medium supplemented with zinc (50 µM) for 7 days. Afterwards mRNA was isolated, used for measuring expression of ZnT1 (**a**), ZnT4 (**b**), ZnT5 (**c**) and ZnT9 (**d**) mRNA expression and normalized to EF1A mRNA. Mean values + SEM of n = 7 independent experiments are shown. Significantly different data sets as determined by one-way Anova and Tukey’s post hoc test do not share the same letters (*p* < 0.05).

Similar to the observations for the transporters of the Zip family, no significant difference in the effects of ZnSO_4_ compared to ZnAA on the expression of ZnTs was found (data not shown). Thus, data were pooled and denoted as ZS as well. Zinc supplementation increased the expression of ZnT1 compared to ZD and ZA cells (Figure 3a), whereas zinc deficiency increased the expression of ZnT4 and ZnT9 compared to ZA and ZS cells (Figure 3b,d).

Only negligible zinc-dependent changes were detected for ZnT5 expression. No homologous sequence was found for ZnT3 and ZnT10. The mRNA expression of ZnT2, ZnT6, ZnT7 and ZnT8 was below the detection limit (Table 2).

### 3.3. Effect of Zinc Status on the Expression of Redox-Related Mediators

**Figure 4:**
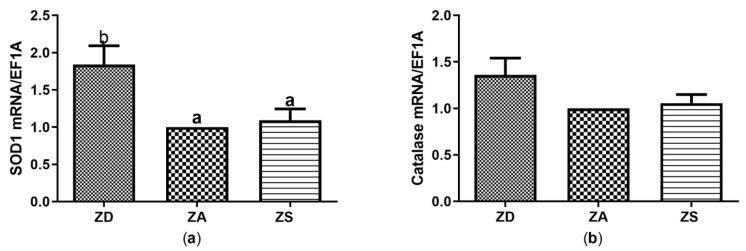
SF cells were cultured in CHELEX-treated medium, zinc adequate (ZA) medium or medium supplemented with zinc (50 µM) for 7 days. Afterwards mRNA was isolated, used for measuring expression of SOD1 (**a**) and Catalase (**b**) mRNA expression and normalized to EF1A mRNA. Mean values + S.E.M. of n = 7 independent experiments are shown. Significantly different data sets as determined by one-way Anova and Tukey’s post hoc test do not share the same letters (*p* < 0.05).

Zinc is generally known for its anti-oxidant function, which has so far not been investigated for the Asian sea bass. While no significant effects of zinc supplementation (ZS) with either ZnSO_4_ or ZnAA on the expression of redox-related enzymes were found in comparison to ZA cells, zinc deficiency caused a significant increase in SOD1 transcription in comparison to ZA and ZS cells (Figure 4a). Catalase expression showed the same trends but the differences between the treatments were not statistically significant (Figure 4b). Glutathione peroxidases 2, 3 and 7 were tested as well, but no expression of those genes was detected in SF cells (data not shown).

### 3.4. Expression of Growth-Related Factors Is Affected by Zinc Deficiency

**Figure 5:**
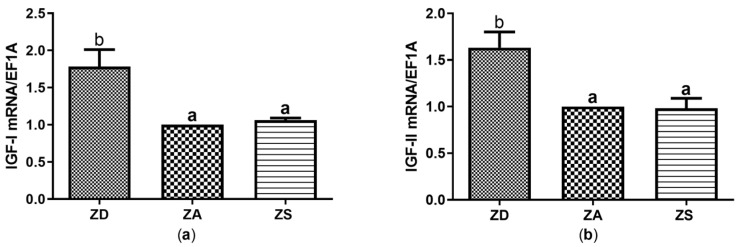
SF cells were cultured in CHELEX-treated medium, zinc adequate (ZA) medium or medium supplemented with zinc (50 µM) for 7 days. Afterwards mRNA was isolated, used for measuring expression of IGF-I (**a**) and IGF-II (**b**) mRNA expression and normalized to EF1A mRNA. Mean values + S.E.M. of n = 7 independent experiments are shown. Significantly different data sets as determined by one-way Anova and Tukey’s post hoc test do not share the same letters (*p* < 0.05).

Similar to what was observed for genes involved in regulating the redox metabolism, zinc supplementation did not significantly alter the expression of IGF-I or IGF-II in comparison to ZA cells. However, zinc deficiency significantly induced the expression of both IGF-I and IGF-II compared to zinc adequate control cells (Figure 5a,b).

### 3.5. Metabolism Is Affected by the Zinc Status

**Figure 6:**
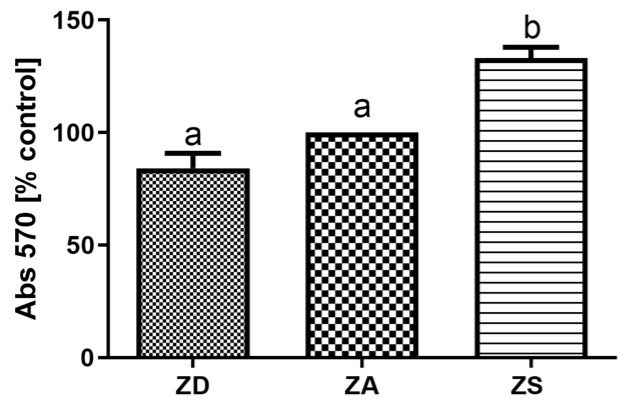
SF cells were cultured in CHELEX-treated medium, zinc adequate (ZA) medium or medium supplemented with zinc (50 µM) for 7 days. Afterwards the MTT test was performed. Mean values + S.E.M. of 8 replicates per treatment and n = 3 independent experiments are shown. Significantly different data sets as determined by one-way Anova and Tukey’s post hoc test do not share the same letters (*p* < 0.05).

To obtain a first impression on the effects of the extracellular zinc availability on the metabolism of the cells, the MTT test was performed. As illustrated in Figure 6, cells cultured in zinc deficient medium tended to have a reduced metabolic activity, but this was not significantly different from ZA cells, whereas zinc supplementation significantly increased metabolic activity in comparison to ZA cells.

## 4. Discussion

The uptake of zinc by cells may depend not only on the nature of the zinc compound used, but also on the species and cell type investigated as well as the overall composition of the diet [27]. While zinc transporters are largely responsible for the transport of zinc across cellular membranes in mammalian cells, data on zinc transport into fish cells is rather scarce and mostly limited to investigations on zebrafish and catfish [18,20]. Our general knowledge on the effects of alterations in zinc availability on the metabolism of fish cells at the molecular level is also limited but would be helpful to improve growth and health of fish in aquaculture. In the current study, we thus addressed these open questions using epithelial SF cells derived from Asian sea bass. This fish type is frequently used for commercial fishing and aquaculture and is thus very important economically.

Intracellular free zinc levels were increased more rapidly and to a greater extent by ZnAA compared to ZnSO_4_, when analyzed in the short term within the first 30 min after supplementation had started (Figure 1a). However, in contrast, no differences between the two supplements were observed when total cellular zinc and the expression of *MT* were analyzed after supplementation for 7 days (Figure 1b,c). In agreement with the latter result for SF cells, no differences in final zinc levels after the uptake of ZnAA compared to ZnSO_4_ by Asian sea bass where found in vivo [3]. Several studies indicate that FluoZin3-AM accumulates in certain cellular compartments depending on the cell type and that the probe does not necessarily remain in the cytoplasm [24,28,29]. Thus, although results show clear differences in the uptake of the two zinc compounds, this may be true for only distinct compartments, while in other parts of the cell, conditions may be different. Alternatively, uptake of inorganic zinc compounds may require de novo expression or intracellular re-distribution of zinc transporters or zinc-binding proteins and may thus be delayed compared to ZnAA sources [9]. However, and most importantly, supplementation with either ZnSO_4_ or ZnAA was able to significantly elevate zinc levels within the cells (Figure 1), as was also described for RTgutGC (Rainbow Trout gut Guelph Canada) cells and cells derived from common carp [27,30]. As expected, intracellular zinc levels decreased in SF cells grown in zinc deficient medium.

Expression of some, but not of all, zinc transporters and zinc-binding proteins appear to be affected by changes in zinc homeostasis. Moreover, Zips and ZnTs exhibit tissue-specific expression and distribution [9,18]. In SF cells, we detected mRNA expression of MT and 11 zinc transporters. The expressions of MT, ZnT1, ZnT4, ZnT9, Zip4, Zip6, Zip10, Zip14 were affected by changes in zinc homeostasis (Figure 1c, Figure 2 and Figure 3). MT expression was increased by both zinc supplements and was decreased by zinc deficiency (Figure 1c), which is in line with reports from various studies in mammals and fish [31,32]. Interestingly, ZnT1 mRNA levels have been shown to be directly regulated by zinc across various cell types and species including zebrafish gill and zebrafish intestine [18,30,31,32]. This indicates that ZnT1 may be a highly conserved transporter, exporting zinc to maintain zinc homeostasis in cells during high zinc conditions [32,33]. Down-regulation of ZnT1 expression during zinc deficiency has been described for zebrafish [18]. We also found a slight but not significant decrease in ZnT1 expression in zinc deficient SF cells (Figure 3a). On the other hand, zinc deficiency increased the expression of ZnT4 and ZnT9 in SF cells (Figure 3b,d). For ZnT4, up- and down-regulation by zinc deficiency were reported for rat jejunum and a human osteoblastic cell line, respectively [34,35]. ZnT9 mRNA was upregulated in zinc deficient human osteoblastic cells, as observed here [35] and the role of ZnT9 in maintaining zinc homeostasis is accepted [36]. Probably to facilitate or hinder zinc uptake, mRNA expression of Zip4, Zip6, Zip10 and Zip14 was upregulated in zinc deficient SF cells, while zinc supplementation reduced expression of Zip10. Zip10 expression was also upregulated in zinc deficient zebrafish together with Zip3 [37]. Zip3 expression was also slightly upregulated in zinc deficient SF cells (Figure 2g). Zinc supplementation affected the expressions of Zip1, Zip4 and Zip8 in zebrafish [38]. Regarding epithelial cells from yellow catfish, Zip1, Zip3, Zip6, Zip7, Zip8, Zip9, Zip10, Zip11, Zip13 and Zip14 appeared to be relevant for maintaining zinc homeostasis, depending on the duration and extent of changes in extracellular zinc [20,33]. Zip10 thus seems to be a common zinc-sensitive transporter in various species, while zinc-dependent regulation of other zinc transporters may strongly depend on cell type, zinc status, duration of zinc deficiency or supplementation and the species investigated.

Although only mRNA data are presented, our results indicate that zinc uptake, regulation of intracellular zinc levels and adjustment to extracellular zinc conditions by SF cells are similar to what has been described for mammals. However, it needs to be tested, whether localization and function of MT and the zinc transporters in SF cells and for example human epithelial cells are the same. Restricting our analysis to mRNA expression is a clear limitation of this study. Since the data shown for zinc transporters and MT presented here (Figure 1, Figure 2 and Figure 3) match well with major findings regarding zinc metabolism in other species [9,19,31,39], the validity of our data is still probably high. Unfortunately, the available resources for studying zinc transporters and zinc-related processes in Asian sea bass on a molecular level are so far limited. For example, we were not able to find sea bass-specific antibodies for zinc transporters. However, we hope that our data will increase the attractivity of Asian sea bass for zinc-related research and thus the development of antibodies in the future.

Zinc is well known to have anti-oxidant properties and to support cell growth as well as proliferation [9]. However, our data revealed no significant effects of zinc supplementation on the mRNA expression of CAT, SOD, IGF-I or IGF-II (Figure 4 and Figure 5). However, contrasting effects of zinc supplementation on expression and activity of all four factors have been described elsewhere for other species. Thus, zinc supplementation of coho salmon, olive flounders and other fish improved the activity of anti-oxidant enzymes, including SOD and CAT [10,40], and activities of SOD and CAT in serum, intestine, liver and muscle tissue of juvenile Jian carp increased after zinc supplementation [41]. Investigation of zinc-treated disk abalone revealed that the transcriptional responses of SOD genes were quite variable depending on the tissues and isoforms investigated [42]. Finally, zinc plus lead supplementation of carp increased SOD and CAT activity after 24 h of supplementation. In contrast, activities of both enzymes were decreased after several days of zinc and lead treatment [43]. Although zinc was combined with lead in that study, the time-dependency of the effects may explain some of the discrepancies between our data and existing studies. Similarly, effects of zinc supplementation on IGF-I and IGF-II expression strongly varied depending on species, dosage and duration of zinc treatment. Supplementation of zebrafish with moderate concentrations of zinc chloride (ZnCl_2_) or zinc oxide nanoparticles did not affect growth or IGF-I mRNA expression, while treatment with high ZnCl_2_ concentrations decreased both parameters in zebrafish [44,45]. Zinc supplementation of rainbow trout white muscle cells caused a decrease in IGF mRNA levels as well [7].

A lack of zinc induces a stress response in various cell types. Amongst others, production of reactive oxygen species is induced and growth retardation, maldevelopment and dwarfism in fish have been reported [11]. To counteract oxidative stress, anti-oxidatively active enzymes can be induced, as observed here in zinc deficient SF cells (Figure 4) [40]. However, several studies indicate that long-term feeding of a zinc deficient diet to animals such as young grass carp reduces the *SOD* and *CAT* expression and activity in various tissues [8,46]. In contrast, CAT activity was increased in the heart of zinc deficient rats and a slight increase in the activity of SOD was observed as well [47]. Embryos from zinc deficient ducks revealed higher expression and activities of CAT [39]. Moreover, thermal or nutritional stress significantly increased SOD mRNA and CAT levels in the tropical fish species *Chanos chanos* [48] and in plasma of Starry flounder [49]. This is in line with our data for zinc deficiency-induced stress. We were surprised that zinc deficiency significantly increased mRNA expression of IGF-I and IGF-II (Figure 5). In fact, most studies in mammals revealed a decrease in IGF mRNA expression during zinc deficiency in various tissues [50]. On the other hand, no changes in IGF mRNA expression were found in zinc deficient rat liver cells in vitro. Furthermore, increased expression of IGF-I mRNA in the liver of zinc deficient weanling rats was described, while serum IGF-I levels remained constant [51]. Interestingly, effects of starvation and refeeding on IGF-I levels were slower and restricted to the liver in salmon compared to rats. This indicates that there may be large species-dependent differences in IGF-I expression as well as in the adaptation to food deprivation [52].

The available studies on effects of changes in zinc homeostasis on the expression and activity of anti-oxidant mediators and IGF are difficult to compare due to high heterogeneity. Large differences exist between the chosen (fish) species, supplementation in vivo or in vitro, the zinc dose, the zinc compound and the duration of supplementation. In addition, complete fish or organs were often analyzed and enzyme activity rather than mRNA expression of SOD and CAT was assessed. Thus, differences in experimental setups may explain the contrasting data. Furthermore, ectothermic animals such as Asian sea bass are known to generally have a slower metabolism and many fish are metabolically adapted to extended periods of food deprivation [52]. Thus, the zinc-deficient cells may still be capable of mounting an anti-oxidative and IGF response within the first 7 days of zinc deficiency. However, since metabolic activity was reduced in zinc-deficient SF cells (Figure 6), they may overall react to zinc deficiency with a stress response. The induction of anti-oxidative factors (Figure 4) may overall not rescue the cells from zinc deficiency-induced apoptosis, In summary, our data indicate that zinc deficiency increases the expression of CAT, SOD, IGF-I and IGF-II in SF epithelial cells (Figure 4 and Figure 5), which remains to be tested on the protein and enzyme activity level. Moreover, the activity of those factors needs to be assessed to test if our findings are relevant for the zinc-regulated anti-oxidative response of epithelial cells.

## 5. Conclusions

It can be concluded that our data provide essential basic information on mRNA levels indicating expression of genes relevant for zinc metabolism in cells from the Asian sea bass under different zinc supplementation regimes. Those data may enable future studies focusing on the molecular mechanisms underlying zinc’s effect on the growth and the redox response of Asian sea bass. Parallels found in the expression of zinc transporters and MT between mammalian and Asian sea bass cells were found. Thus, Asian sea bass, or cultures of Asian sea bass cells, may provide valuable models to address zinc-related questions in fish in general.

## Figures and Tables

**Table 1:** Primer sequences.

Gene	Forward Primers	Reverse Primers	Source
EF1A	GTTGCCTTTGTCCCCATCTC	CTTCCAGCAGTGTGGTTCCA	[2]
IGF-I	AAGACTAAGGCAGCTCGCTC	CTTGTCCACTTTGTGCCCTG	^1^XM_018697285.1
IGF-II	TGCAGAGACGCTGTGTGG	GCCTACTGAAATAGAAGCCTCTGT	[25]
MT	CACCTGCACAACTGCTCCTG	ACGCAGCCTGAGGCACAC	[26]
Catalase	CCCACTTTGACAGGGAACGA	AACACCTTGGCCTTGCAGTA	^1^XM_018675907.1
SOD1	GTGATCCATGAGAAGGCCGA	GCGATGCCAATGACTCCACA	^1^XM_018691152.1
Zip1	CCACCACGTCCATGTTGACT	GGCCAATAGCAAGACCCTCA	^1^XM_018678733.2
Zip2	TCCGCAGATCGTCCTCCTAA	GCTGTACTGTGAGCGGTGTT	^1^XM_018681747.2
Zip3	CAGAGACAAGGTGGGCGATG	GGTGAGGAAGAAACCGAGCA	^1^XM_051068639.1 ^1^XM_018662136.2 ^1^XM_018662135.2 ^1^XM_018690367.2 ^1^XM_018690365.2
Zip4	GACTGGCTGATATGCTCCCC	CATCCGGTTAGAAGGCCGAC	^1^XM_018670744.2
Zip6	CGCATCACCAGAAACACGTC	CGGGTTCTCCGTCTTCTTCC	^1^XM_018674935.2 ^1^XM_018674937.2 ^1^XM_018674936.2 ^1^XM_018674938.2
Zip7	ACATGCTCTGGGTAGCCCC	CATGCGAGTGTCCGTGAGAG	^1^XM_018682170.2
Zip8	GCTCGGCCTTTTGCTGATAC	CGTTGGAGAAGAGTGTCCCG	^1^XM_018666273.2
Zip9	CAGGGACATTTGCTGGCCTT	AGAAGAGCATTCCCACACCTG	^1^XM_018681638.2
Zip10	TGCCCATCCTCAACCAATCC	GGCAGCAGGTGTAGTAGAGC	^1^XM_018689799.2 ^1^XM_018683164.2
Zip11	GTTCACCTGGGGTCTAACCG	GGGTCCAGCTCTACCTATCCT	^1^XM_018703772.2 ^1^XM_051066206.1
Zip12	CCCGGTAGGAACATGCACTT	GGGAAGACCCAGTGCTTTGA	^1^XM_018693642.1
Zip13	CACTCAGAAACGGGCACCAT	AAGGTGGTGAGGAACCCAAC	^1^XM_018686288.2
Zip14	GTTGTTTGTTGAGCGGAGTCG	GCTTCTTCACTGGCTGGAGG	^1^XM_018688493.2 ^1^XM_018688494.2 ^1^XM_018688494.2
ZnT1	CCACCATCCAGCCAGAGTTT	AGCCTGTACCCTGTTTGTCG	^1^XM_018694997.2
ZnT2	CACGAGAGGAATGCCAAGATGT	CGGCCATGCCTCCGTTTTTA	^1^XM_018687330.2
ZnT4	TGACCGACGCTTTGCACATA	AGACCAGTATGGCCGTCAGA	^1^XM_018683556.2 ^1^XM_018683555.2
ZnT5	GCCATCTGCAACGCAAAGAT	CGGGTGAGTCCAGAAGTTGG	^1^XM_051072778.1
ZnT6	ATGCAGGCAGGGGTGC	TAGCGGGTTGCTAGTTCCAA	^1^XM_018660807.2 ^1^XM_018660809.2
ZnT7	CATCGGGACGTTAAAGCTGC	ACATAGAGCTGTCGAACCCC	^1^XM_018662649.2
ZnT8	GACCGCGAACGAGAGAAGAA	CCACCGAGGATTTCACCGAT	^1^XM_018702603.2
ZnT9	AGAGCGTCCCTATCACAACG	GAAGGCTGAACCACAGGCTA	^1^XM_018694108.1 ^1^XM_018694109.1

^1^ NCBI-RefSeq.

**Table 2:** Reference Sequences (RefSeq) for all Zip and ZnT mRNAs that were below the detection limit in SF cells. Genes, where no homologous sequences were found for Asian sea bass are indicated as well.

Transporter	RefSeq	Zinc Effect
Zip1	XM_018678733.2	No mRNA expression detected
Zip5	No homologous sequence found	
Zip7	XM_018682170.2	No mRNA expression detected
Zip9	XM_018681638.2	No mRNA expression detected
Zip11	XM_018703772.2 XM_051066206.1	No mRNA expression detected
Zip12	XM_018693642.1	No mRNA expression detected
Zip13	XM_018686288.2	No mRNA expression detected
ZnT2	XM_018687330.2	Not detected
ZnT3	No homologous sequence found	
ZnT6	XM_018660807.1	Not detected
ZnT7	XM_018662649.2	Not detected
ZnT8	XM_018702603.2	Not detected
ZnT10	No homologous sequence found	

## Data Availability

The authors confirm that all relevant data supporting the findings of this study are available within the article. Additional data that support the findings of this study are available on request from the corresponding author [I.W.].

## References

[1] Béné C., Barange M., Subasinghe R., Pinstrup-Andersen P., Merino G., Hemre G.-I., Williams M. (2015). Feeding 9 billion by 2050—Putting fish back on the menu. Food Sec..

[2] Paria A., Dong J., Babu P.P.S., Makesh M., Chaudhari A., Thirunavukkarasu A.R., Purushothaman C.S., Rajendran K.V. (2016). Evaluation of candidate reference genes for quantitative expression studies in Asian seabass (*Lates calcarifer*) during ontogenesis and in tissues of healthy and infected fishes. Indian J. Exp. Biol..

[3] Sansuwan K., Jintasataporn E.-O., Chumkam S. (2019). Effects of Dietary Zinc Amino Acid Complex and Zinc Sulfate on Growth Performance, Digestive Enzyme Activity and Immune Response in Asian Seabass (*Lates calcarifer*). J. Aquac. Res. Dev..

[4] Samat N.A., Yusoff F.M., Rasdi N.W., Karim M. (2020). Enhancement of Live Food Nutritional Status with Essential Nutrients for Improving Aquatic Animal Health: A Review. Animals.

[5] Ellis S.C., Reigh R.C. (1991). Effects of dietary lipid and carbohydrate levels on growth and body composition of juvenile red drum, Sciaenops ocellatus. Aquaculture.

[6] Lin S., Lin X., Yang Y., Li F., Luo L. (2013). Comparison of chelated zinc and zinc sulfate as zinc sources for growth and immune response of shrimp (*Litopenaeus vannamei*). Aquaculture.

[7] Ekinci D., Ceyhun S.B., Aksakal E., Erdoğan O. (2011). IGF and GH mRNA levels are suppressed upon exposure to micromolar concentrations of cobalt and zinc in rainbow trout white muscle. Comp. Biochem. Physiol. Part C Toxicol. Pharmacol..

[8] Huang F., Jiang M., Wen H., Wu F., Liu W., Tian J., Yang C. (2015). Dietary zinc requirement of adult Nile tilapia (*Oreochromis niloticus*) fed semi-purified diets, and effects on tissue mineral composition and antioxidant responses. Aquaculture.

[9] Wessels I., Fischer H.J., Rink L. (2021). Dietary and Physiological Effects of Zinc on the Immune System. Annu. Rev. Nutr..

[10] Min B.-H., Saravanan M., Nam S.-E., Eom H.-J., Rhee J.-S. (2019). Waterborne zinc pyrithione modulates immunity, biochemical, and antioxidant parameters in the blood of olive flounder. Fish Shellfish Immunol..

[11] National Research Council (2011). Nutrient Requirements of Fish and Shrimp.

[12] Tan B., Mai K. (2001). Zinc methionine and zinc sulfate as sources of dietary zinc for juvenile abalone, Haliotis discus hannai Ino. Aquaculture.

[13] Paripatananont T., Lovell R.T. (1995). Responses of Channel Catfish Fed Organic and Inorganic Sources of Zinc to Edwardsiella ictaluri Challenge. J. Aquat. Anim. Health.

[14] Apines-Amar M.J.S., Satoh S., Caipang C.M.A., Kiron V., Watanabe T., Aoki T. (2004). Amino acid-chelate: A better source of Zn, Mn and Cu for rainbow trout, Oncorhynchus mykiss. Aquaculture.

[15] Buentello J.A., Goff J.B., Gatlin D.M. (2009). Dietary zinc requirement of hybrid striped bass, *Morone chrysops* × *Morone saxatilis*, and bioavailability of two chemically different zinc Compounds. J. World Aquac. Soc..

[16] do Carmo e Sá M.V., Pezzato L.E., Ferreira Lima M.M.B., de Magalhães Padilha P. (2004). Optimum zinc supplementation level in Nile tilapia *Oreochromis niloticus* juveniles diets. Aquaculture.

[17] Ma R., Hou H., Mai K., Bharadwaj A.S., Ji F., Zhang W. (2014). Comparative study on the bioavailability of chelated or inorganic zinc in diets containing tricalcium phosphate and phytate to turbot (*Scophthalmus maximus*). Aquaculture.

[18] Zheng D., Feeney G.P., Handy R.D., Hogstrand C., Kille P. (2014). Uptake epithelia behave in a cell-centric and not systems homeostatic manner in response to zinc depletion and supplementation. Metallomics.

[19] Maret W., Jacob C., Vallee B.L., Fischer E.H. (1999). Inhibitory sites in enzymes: Zinc removal and reactivation by thionein. Proc. Natl. Acad. Sci. USA.

[20] Chen S.-W., Wu K., Lv W.-H., Song C.-C., Luo Z. (2020). Molecular characterization of ten zinc (Zn) transporter genes and their regulation to Zn metabolism in freshwater teleost yellow catfish Pelteobagrus fulvidraco. J. Trace Elem. Med. Biol..

[21] Ho E., Dukovcic S., Hobson B., Wong C.P., Miller G., Hardin K., Traber M.G., Tanguay R.L. (2012). Zinc transporter expression in zebrafish (*Danio rerio*) during development. Comp. Biochem. Physiol. Part C Toxicol. Pharmacol..

[22] Chang S., Ngoh G., Kueh L., Qin Q., Chen C., Lam T., Sin Y. (2001). Development of a tropical marine fish cell line from Asian seabass (*Lates calcarifer*) for virus isolation. Aquaculture.

[23] Mayer L.S., Uciechowski P., Meyer S., Schwerdtle T., Rink L., Haase H. (2014). Differential impact of zinc deficiency on phagocytosis, oxidative burst, and production of pro-inflammatory cytokines by human monocytes. Metallomics.

[24] Rolles B., Maywald M., Rink L. (2021). Intracellular zinc during cell activation and zinc deficiency. J. Trace Elem. Med. Biol..

[25] Betancor M.B., Caballero M.J., Terova G., Saleh R., Atalah E., Benítez-Santana T., Bell J.G., Izquierdo M. (2012). Selenium inclusion decreases oxidative stress indicators and muscle injuries in sea bass larvae fed high-DHA microdiets. Br. J. Nutr..

[26] Thanomsit C., Nantanawat P., Wassmur B., Gräns J., Celander M.C., Kanchanopas-Barnette P. (2013). Characterization of Metallothionein from Asian Sea Bass (*Lates calcarifer*, Bloch) and Application as a Biomarker for Heavy Metal Exposure in Thailand. Asian J. Water Environ. Pollut..

[27] Prabhu P.A.J., Stewart T., Silva M., Amlund H., Ørnsrud R., Lock E.-J., Waagbo R., Hogstrand C. (2018). Zinc uptake in fish intestinal epithelial model RTgutGC: Impact of media ion composition and methionine chelation. J. Trace Elem. Med. Biol..

[28] Han Y., Goldberg J.M., Lippard S.J., Palmer A.E. (2018). Superiority of SpiroZin2 Versus FluoZin-3 for monitoring vesicular Zn^2+^ allows tracking of lysosomal Zn^2+^ pools. Sci. Rep..

[29] Kaltenberg J., Plum L.M., Ober-Blöbaum J.L., Hönscheid A., Rink L., Haase H. (2010). Zinc signals promote IL-2-dependent proliferation of T cells. Eur. J. Immunol..

[30] Muylle F., Robbens J., de Coen W., Timmermans J.-P., Blust R. (2006). Cadmium and zinc induction of ZnT-1 mRNA in an established carp cell line. Comp. Biochem. Physiol. Part C Toxicol. Pharmacol..

[31] Liuzzi J.P., Blanchard R.K., Cousins R.J. (2001). Differential regulation of zinc transporter 1, 2, and 4 mRNA expression by dietary zinc in rats. J. Nutr..

[32] Balesaria S., Hogstrand C. (2006). Identification, cloning and characterization of a plasma membrane zinc efflux transporter, TrZnT-1, from fugu pufferfish (*Takifugu rubripes*). Biochem. J..

[33] Chen G.-H., Luo Z., Wei C.-C., Li D.-D., Pan Y.-X. (2018). Six indicator genes for zinc (Zn) homeostasis in freshwater teleost yellow catfish Pelteobagrus fulvidraco: Molecular characterization, mRNA tissue expression and transcriptional changes to Zn exposure. Biometals.

[34] Pfaffl M.W., Windisch W. (2003). Influence of zinc deficiency on the mRNA expression of zinc transporters in adult rats. J. Trace Elem. Med. Biol..

[35] Alluri K., Nair K.P.M., Kotturu S.K., Ghosh S. (2020). Transcriptional Regulation of Zinc Transporters in Human Osteogenic Sarcoma (Saos-2) Cells to Zinc Supplementation and Zinc Depletion. Biol. Trace Elem. Res..

[36] Song C.-C., Wu L.-X., Chen G.-H., Lv W.-H., Chen S.-W., Luo Z. (2020). Six members of SLC30A/ZnTs family related with the control of zinc homeostasis: Characterization, mRNA expression and their responses to dietary ZnO nanoparticles in yellow catfish. Aquaculture.

[37] Zheng D., Feeney G.P., Kille P., Hogstrand C. (2008). Regulation of ZIP and ZnT zinc transporters in zebrafish gill: Zinc repression of ZIP10 transcription by an intronic MRE cluster. Physiol. Genom..

[38] Puar P., Niyogi S., Kwong R.W.M. (2020). Regulation of metal homeostasis and zinc transporters in early-life stage zebrafish following sublethal waterborne zinc exposure. Aquat. Toxicol..

[39] Gao W., Huang L., Zhang X., Ma X., Wang W., Zheng Y., Geng W., Liu C., Wei S., Yang L. (2021). Effect of Maternal Marginal Zinc Deficiency on Development, Redox Status, and Gene Expression Related to Oxidation and Apoptosis in an Avian Embryo Model. Oxid. Med. Cell. Longev..

[40] Yu H.-R., Li L.-Y., Shan L.-L., Gao J., Ma C.-Y., Li X. (2021). Effect of supplemental dietary zinc on the growth, body composition and anti-oxidant enzymes of coho salmon (*Oncorhynchus kisutch*) alevins. Aquac. Rep..

[41] Feng L., Tan L.-N., Jiang J., Jiang W.-D., Hu K., Li S.-H., Zhou X.-Q. (2011). Influence of dietary zinc on lipid peroxidation, protein oxidation and antioxidant defence of juvenile Jian carp (*Cyprinus carpio* var. Jian). Aquac. Nutr..

[42] Kim K.-Y., Lee S.Y., Cho Y.S., Bang I.C., Kim K.H., Kim D.S., Nam Y.K. (2007). Molecular characterization and mRNA expression during metal exposure and thermal stress of copper/zinc- and manganese-superoxide dismutases in disk abalone, Haliotis discus discus. Fish Shellfish Immunol..

[43] St. Dimitrova M., Tishinova V., Velcheva V. (1994). Combined effect of zinc and lead on the hepatic superoxide dismutase-catalase system in carp (*Cyprinus carpio*). Comp. Biochem. Physiol. Part C Pharmacol. Toxicol. Endocrinol..

[44] Horie Y., Yonekura K., Suzuki A., Takahashi C. (2020). Zinc chloride influences embryonic development, growth, and Gh/Igf-1 gene expression during the early life stage in zebrafish (*Danio rerio*). Comp. Biochem. Physiol. Part C Toxicol. Pharmacol..

[45] Fasil D.M., Hamdi H., Al-Barty A., Zaid A.A., Parashar S.K.S., Das B. (2021). Selenium and Zinc Oxide Multinutrient Supplementation Enhanced Growth Performance in Zebra Fish by Modulating Oxidative Stress and Growth-Related Gene Expression. Front. Bioeng. Biotechnol..

[46] Song Z.-X., Jiang W.-D., Liu Y., Wu P., Jiang J., Zhou X.-Q., Kuang S.-Y., Tang L., Tang W.-N., Zhang Y.-A. (2017). Dietary zinc deficiency reduced growth performance, intestinal immune and physical barrier functions related to NF-κB, TOR, Nrf2, JNK and MLCK signaling pathway of young grass carp (*Ctenopharyngodon idella*). Fish Shellfish Immunol..

[47] Betteger W.J., Bray T.M. (1989). Effect of dietary zinc or copper deficiency on catalase, glutathione peroxidase and superoxide dismutase activities in rat heart. Nutr. Res..

[48] Chang C.-H., Mayer M., Rivera-Ingraham G., Blondeau-Bidet E., Wu W.-Y., Lorin-Nebel C., Lee T.-H. (2021). Effects of temperature and salinity on antioxidant responses in livers of temperate (*Dicentrarchus labrax*) and tropical (*Chanos Chanos*) marine euryhaline fish. J. Therm. Biol..

[49] Kim S.-S., Han G.-S., Yoo H.-K., Kim K.-T., Byun S.-G., Jung M.-M., Kim W.-J., Hwang S.-D. (2021). Effect of Temperature Fluctuation and Nutritional Status on Starry Flounder, *Platichthys stellatus*, Survival and Adaptive Physiological Response. JMSE.

[50] Ninh N.X., Maiter D., Lause P., Chrzanowska B., Underwood L.E., Ketelslegers J.M., Thissen J.P. (1998). Continuous administration of growth hormone does not prevent the decrease of IGF-I gene expression in zinc-deprived rats despite normalization of liver GH binding. Growth Horm. IGF Res..

[51] Hall A.G., Kelleher S.L., Lönnerdal B., Philipps A.F. (2005). A graded model of dietary zinc deficiency: Effects on growth, insulin-like growth factor-I, and the glucose/insulin axis in weanling rats. J. Pediatr. Gastroenterol. Nutr..

[52] Duan C. (1998). Nutritional and developmental regulation of insulin-like growth factors in fish. J. Nutr..

